# Vertical margin distance in T1 colorectal carcinoma resected by endoscopic submucosal dissection affects prognosis after additional surgery

**DOI:** 10.1007/s00384-024-04700-0

**Published:** 2024-08-16

**Authors:** Fumiaki Tanino, Ken Yamashita, Shinji Nagata, Toshio Kuwai, Yuki Kamigaichi, Hidenori Tanaka, Yuzuru Tamaru, Hidehiko Takigawa, Naoki Asayama, Yuji Urabe, Fumio Shimamoto, Shiro Oka

**Affiliations:** 1 Department of Gastroenterology, Hiroshima University Hospital, 1-2-3 Kasumi, Minami-Ku, Hiroshima, 734-8551 Japan; 2 Department of Gastroenterology, Hiroshima City North Medical Center Asa Citizens Hospital, Hiroshima, Japan; 3 Department of Endoscopy, National Organization Kure Medical Center and Chugoku Cancer Center, Kure, Japan; 4 Department of Health Sciences, Hiroshima Cosmopolitan University, Hiroshima, Japan

**Keywords:** Colorectal cancer, Metastasis, Recurrence, Vertical margin, Endoscopic submucosal dissection

## Abstract

**Purpose:**

A vertical margin (VM) distance of < 500 µm is a risk factor for recurrence in patients with T1 colorectal carcinoma (CRC) resected by endoscopy. We aimed to determine the effects of the VM distance on the recurrence and prognosis of T1 CRC.

**Methods:**

We enrolled 168 patients with T1 CRC who underwent additional surgery after endoscopic submucosal dissection (ESD) at multiple centers between 2008 and 2016. None of the patients were followed up for < 5 years. The enrolled 168 patients were classified into patients with VM distance of < 500 µm including positive VM (*n* = 72 [43%], VM distance < 500 µm group) and patients with VM distance of ≥ 500 µm (*n* = 96 [57%], VM distance ≥ 500 µm group). The clinicopathological features, recurrence rates, and prognoses were compared between the groups using propensity-score matching (PSM).

**Results:**

Tumors recurred in eight of the 168 patients (5%) with VM distance < 500 µm. After PSM, the rate of overall recurrence and local recurrence in the VM distance < 500 µm group were significantly higher than those in the VM distance ≥ 500 µm group. The 5-year recurrence-free survival rate was significantly higher in the VM distance ≥ 500 µm group than that in VM distance < 500 µm group after PSM (100% vs. 89%, *p* < 0.012).

**Conclusions:**

Complete en bloc resection of T1 CRC via ESD must include a sufficient amount of SM to reduce the risk of metastasis and recurrence after additional surgery.

**Supplementary Information:**

The online version contains supplementary material available at 10.1007/s00384-024-04700-0.

## Introduction 

Colorectal carcinoma (CRC) is one of the most common malignancies of the gastrointestinal tract worldwide [1]. Intramucosal carcinoma does not metastasize to the lymph nodes (LN) and is a good indication for endoscopic resection (ER) [2]. However, surgery with LN dissection is recommended when T1 CRC is suspected as a rule [3].

Preoperative diagnosis of submucosal (SM) invasion depth is important for treatment selection. According to the Japan Society of Cancer for Colon and Rectum (JSCCR) guidelines 2022, risk factors for LN metastasis (LNM) include the depth of SM invasion, histological grade, lymphatic and venous invasion, and tumor budding [3–6]. Additional surgery after ER is recommended when at least one risk factor for LNM is identified [3]. However, the rate of LNM in T1 CRC is 10% [3, 7, 8]. Therefore, additional surgeries after ER for approximately 90% of patients with T1 CRC who do not have LNM might result in overtreatment. The reported rate of LNM is 1.2% in patients with T1 CRC whose only high-risk histological characteristic is an SM invasion depth of ≥ 1000 µm [9]. The number of T1 CRC resected by ER has increased due to society’s advancing age and patient’s comorbidities [10]. Under these circumstances, endoscopic submucosal dissection (ESD) is considered useful for en bloc resection as a total excisional biopsy in patients with clinical T1 CRC [11–13].

The ninth edition of the Japanese Classification of Colorectal, Appendiceal, and Anal Carcinoma describes local recurrence after ER occurred in patients with the distance of < 500 µm between the site of cancer invasion and the vertical margin (VM) [3]. However, the distance that should be maintained from the site of cancer invasion to the VM to reduce the risk of local recurrence in T1 CRC has not been established. We previously evaluated the effects of the distance from the vertical tumor margin to the edge of resected specimen (VM distance) in ER for T1b CRC on the prognosis of patients who underwent surgery after ER [14]. For the purpose of generalization, we aimed to evaluate the effects of the VM distance in ESD for T1b CRC on metastatic recurrence and the prognosis of patients who underwent additional surgery after ESD at multiple centers.

## Methods

### Patients

**Fig. 1:**
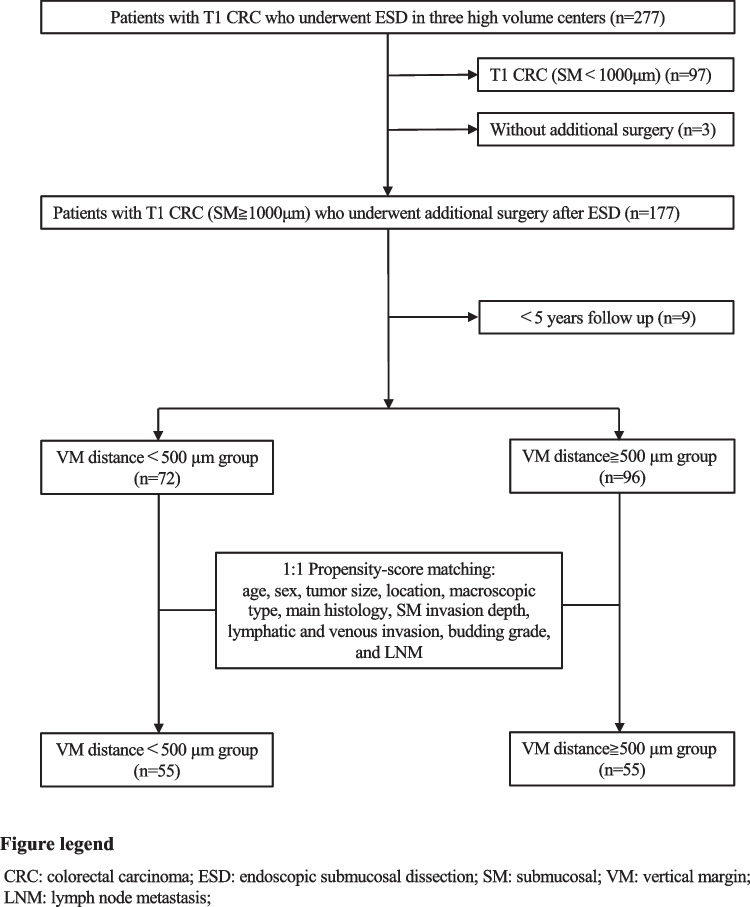
Flow chart of enrolled patients

Figure 1 shows the flow of patients through the study. Among 277 patients with T1 CRC who underwent ESD between January 2008 and June 2016 (at Hiroshima University Hospital, Hiroshima City North Medical Center Asa Citizens Hospital, and National Organization Kure Medical Center and Chugoku Cancer Center), we excluded 109 based on the following: SM invasion depth of < 1000 µm, treated only by ESD, and < 5 years of follow-up. We finally analyzed data from 168 patients. We did not include patients with pedunculated-type CRC because they might be easier to treat by endoscopic mucosal resection than by ESD. Excision of adequate specimens was necessary to evaluate the pathological features. Therefore, only patients who underwent resection using ESD were included. The patients were classified into two groups based on the VM distance of ESD specimens: patients with VM distance < 500 µm including positive VM (VM distance < 500 µm group, *n* = 72 [43%]) and patients with VM distance of ≥ 500 µm (VM distance ≥ 500 µm group, *n* = 96 [57%]). Finally, to align the background factors for each group, we performed one-to-one propensity-score matching (PSM) with age, sex, tumor size, location, macroscopic type, main histology, SM invasion depth, lymphatic invasion, venous invasion, budding grade, and LNM as covariates. Thereafter, we identified 55 patients in each group. All cases were performed ESD by the experienced experts. The study proceeded according to ethical standards of Declaration of Helsinki (2014). The Ethics Committees at Hiroshima University and its affiliated hospital approved the study protocol (Approval No. E-0334) and was in accordance with the guidelines of the Ministry of Health Labour and Welfare. All patients provided informed consent prior to the procedures.

### Indications for ESD

The indications for ER in early-stage CRC followed the JSCCR guidelines [3]. Intramucosal carcinoma and T1 CRC with SM invasion depth of < 1000 µm are at low risk for LNM and a good indication for en bloc resection by ER. By contrast, clinically obvious T1b CRC is usually surgically treated. However, we sometimes attempted ER for T1b CRC as a total excisional biopsy depending on patient preference, comorbidities, and general physical condition. The indications for ESD were as follows: lesions difficult to resect en bloc (non-granular, laterally spreading tumors, particularly those of the pseudo-depressed type), lesions with a type V_I_ pit pattern, carcinoma with shallow SM invasion, large elevated lesions of suspected carcinoma, mucosal lesions with SM fibrosis caused by biopsy or severe peristalsis, local residual early carcinoma after ER, and sporadically localized lesions in chronic inflammation such as ulcerative colitis.

### Indications for additional surgery after ESD

According to the JSCCR guidelines, patients with VM positivity require additional surgery after ESD. Patients with VM negativity are considered for additional surgery if any of the following pathological features are found in resected specimens: SM invasion depth of ≥ 1000 µm, main histology of poorly differentiated adenocarcinoma or mucinous carcinoma or signet-cell carcinoma, positive lymphatic and/or venous invasion, and budding grade 2/3 at the site of deepest invasion [3]. As a general rule, additional surgery should proceed within 3 months of ESD. D2 LN dissection (middle LN) was performed according to the JSCCR guidelines.

### Pathological evaluation

Resected specimens were fixed in 10% buffered formalin for 12–48 h. Surgical and endoscopic specimens were cut into parallel Sects. 3–4- and 2-mm thick, respectively. A single gastrointestinal pathologist diagnosed all histopathological findings, and the patients were blinded to the clinical information. Pathological features, including SM invasion depth, histological grade, tumor budding grade, and lymphatic and venous invasion, were evaluated by hematoxylin–eosin (HE) and specific staining (Victoria blue, Elastica van Gieson, D2-40; Desmin) as needed. The SM invasion depth was measured from the lower border of the muscularis mucosae when possible. If the muscularis mucosae could not be identified or located, SM invasion depth was measured from the surface layer of the mucosa [3, 15]. The histological grade was classified as favorable (tubular or papillary adenocarcinoma) and unfavorable (poorly differentiated adenocarcinoma, mucinous carcinoma, or signet-ring cell carcinoma). The histological type of the invasive front was classified as well, moderately well, moderately poor, and poor. Tumor budding grade was graded per microscopic field at 200 × magnification: low grade: grade 1, 0–4 buds; high grade: grade 2, 5–9 buds; and grade 3, 10 or more buds [16]. VM positivity was defined as the presence of tumors and mucinous components at the VM. VM distance was defined as the distance from the site of the deepest invasion of the cancer to the VM.

### Surveillance after additional surgery

The follow-up was extended to > 5 years after the initial treatment. The patients were postoperatively interviewed and physically examined, underwent blood tests, and were assessed by chest and abdominal computed tomography (CT) every 6 months for the first 3 years, then annually for the next 2 years. As a rule, the patients were postoperatively assessed by total colonoscopy annually for 5 years. Recurrence was confirmed by endoscopy, CT, and other findings. Recurrence at CRC sites and pelvic recurrence in patients with rectal CRC were defined as local. Metastases outside the intestinal tract were defined as distant.

### Investigated variables

We compared the clinicopathological variables of age, sex, tumor size, tumor location, macroscopic type, treatment, metastasis/recurrence, main histology, SM invasion depth, VM distance, lymphatic and venous invasion, budding grade, recurrence, local recurrence, and distant metastasis after additional surgery for T1 CRC between VM distance < 500 and ≥ 500 µm groups before and after PSM. We compared the incidences of recurrence, overall survival (OS; to the day of death from any cause), and recurrence-free survival (RFS; elapsed time from the day of ESD until local and/or distant recurrence was identified) between the groups after PSM.

### Statistical analysis

Data are presented as means ± standard deviation. Between-group differences were analyzed using chi-square or Fisher exact tests. Values with *p* < 0.05 were considered statistically significant. The OS and the RFS were calculated using the Kaplan–Meier method. Significant differences in the baseline clinical characteristics of the patients and the influence of possible confounding factors were adjusted using PSM. Propensity scores were estimated using a logistic regression model that included age, sex, tumor size, location, macroscopic type, main histology, SM invasion depth, lymphatic invasion, venous invasion, budding grade, and LNM as variables. Thereafter, the closest eligible control unit to be paired with each treated unit was selected by one-to-one nearest neighbor matching using a caliper set at 0.25. All data were statistically analyzed using JMP statistical software v. 16.2.0 (SAS Institute, Cary, NC, USA).

## Results

### Baseline characteristics of patients

Table 1 shows the baseline characteristics of the 168 patients. The average age of those enrolled was 69 ± 10 years, and 93 (55%) were male. The mean tumor size was 31 ± 19 mm, and 99 (59%) of 168 lesions were located in the colon. Regarding macroscopic type, 78 (46%) of 168 patients had protruded lesions. VM was positive in 24 (14%) of 168 patients. No patients had local residuals in surgical specimens. LNM after additional surgery was found in 14 (9%) patients. All patients with LNM after additional surgery were included in the VM distance < 500 µm group. Recurrences, local recurrence, and distant metastasis were found in 8 (5%), 4 (2%), and 5 (3%) of the 168 patients, respectively. Table 2 shows the clinicopathological features of patients with T1b CRC before and after PSM. Before PSM, the VM distance < 500 µm group consisted of 72 (43%) patients, and the VM distance ≥ 500 µm group consisted of 96 (57%) patients. Age, sex, tumor location, macroscopic type, and main histology did not significantly differ between the groups. Tumors were significantly smaller in the VM distance < 500 µm group than that in the ≥ 500 µm group (28 ± 19 vs. 33 ± 19 mm; *p* = 0.0422). The SM invasion depth was significantly shallower in the VM distance < 500 µm group than that in the ≥ 500 µm group (2670 ± 1552 vs. 3385 ± 1642 µm; *p* = 0.0023). The rate of lymphatic invasion (31 [43%] vs. 23 [24%]; *p* = 0.0087), venous invasion (30 [42%] vs. 26 [27%]; *p* = 0.0472), budding grade 2/3 (25 [35%] vs. 17 [18%]; *p* = 0.007), and LNM positive (10 [14%] vs. 4 [4%]; *p* = 0.0241) was significantly higher in the VM distance < 500 µm group than that in the VM distance ≥ 500 µm group. The clinicopathological backgrounds did not significantly differ between the groups that were found after PSM.

**Table 1:** Clinicopathological features of enrolled patients (*n* = 168)

Variables		
Age (years, mean ± SD)		69 ± 10
Sex		
Male		93 (55)
Female		75 (45)
Tumor location		
Colon		99 (59)
Rectum		69 (41)
Tumor size (mm)		
Mean ± SD		31 ± 19
Median (range)		25 (5–100)
Macroscopic type		
Protruded	0-Is	49 (29)
	0-Is + IIa	3 (2)
	0-Is + IIc	7 (4)
	0-Isp	17 (10)
	0-Isp + IIc	2 (1)
Superficial	0-IIa	54 (32)
	0-IIa + IIc	35 (21)
	0-IIc	1 (1)
Histology		
Tub/pap		165 (98)
Por/muc/sig		3 (2)
VM		
Negative		144 (86)
Positive		24 (14)
VM distance (µm)		
< 500 (including VM positive)		72 (43)
≧500		96 (57)
Lymphatic invasion positive		54 (32)
Venous invasion positive		56 (33)
Budding grade 2/3		42 (25)
LNM after additional surgery		14 (9)
Local remnants		0 (0)
Recurrence/metastasis		8 (5)
Local recurrence		4 (2)
Distant metastasis		5 (3)

*SD* standard deviation, *ESD* endoscopic submucosal dissection, *tub* tubular adenocarcinoma, *pap* papillary adenocarcinoma, *por* poorly differentiated adenocarcinoma, *muc* mucinous adenocarcinoma, *sig* signet-ring adenocarcinoma, *SM* submucosal, *VM* vertical margin, *LNM* lymph node metastasis

**Table 2:** Clinicopathological features of T1 CRC patients before and after propensity-score matching

Variables			All patients (*n* = 168)			Propensity-matched patients (*n* = 110)		
			VM distance		*p* Value	VM distance		*p* Value
			< 500 µm (*n* = 72)	≧500 µm (*n* = 96)		< 500 µm (*n* = 55)	≧500 µm (*n* = 55)	
Age (years, mean ± SD)			68 ± 8	70 ± 11	0.6361	68 ± 9	70 ± 11	0.3568
Sex		Male	43 (60)	50 (52)	0.3243	31 (56)	28 (51)	0.5662
		Female	29 (40)	46 (48)		24 (44)	27 (49)	
Tumor size (mm, mean ± SD)			28 ± 19	33 ± 19	0.0422	29.9 ± 19.1	28.5 ± 14.9	0.9043
Tumor location		Colon	45 (63)	54 (56)	0.4151	34 (62)	33 (60)	0.8451
		Rectum	27 (37)	42 (44)		21 (38)	22 (40)	
Macroscopic type	Protruded	0-Is	17 (24)	32 (33)	0.0579	13 (24)	21 (38)	0.2053
		0-Is + IIa	1 (2)	2 (2)		1 (2)	0 (0)	
		0-Is + IIc	5 (7)	2 (2)		5 (9)	1 (2)	
		0-Isp	7 (10)	10 (10)		4 (7)	4 (7)	
		0-Isp + IIc	0 (0)	2 (2)		0 (0)	2 (4)	
	Superficial	0-IIa	19 (26)	35 (36)		16 (29)	16 (29)	
		0-IIa + IIc	22 (31)	13 (14)		16 (29)	11 (20)	
		0-IIc	1 (2)	0 (0)		0 (0)	0 (0)	
SM invasion depth (μm, mean ± SD)			2670 ± 1552	3385 ± 1642	0.0023	2874 ± 1666	2747 ± 1438	0.7716
Main histology		Tub/pap	70 (97)	95 (99)	0.4004	53 (96)	54 (98)	0.5583
		Por/sig/muc	2 (3)	1 (1)		2 (4)	1 (2)	
Lymphatic invasion		Negative	41 (57)	73 (76)	0.0087	36 (65)	37 (67)	0.8401
		Positive	31 (43)	23 (24)		19 (35)	18 (33)	
Venous invasion		Negative	42 (58)	70 (73)	0.0472	38 (69)	40 (73)	0.6746
		Positive	30 (42)	26 (27)		17 (31)	15 (27)	
Budding grade		Grade 1	47 (65)	79 (82)	0.0117	41 (75)	43 (78)	0.6535
		Grade 2/3	25 (35)	17 (18)		14 (25)	12 (22)	
LNM		Negative	62 (86)	92(96)	0.0241	50 (91)	53 (96)	0.2413
		Positive	10 (14)	4 (4)		5 (9)	2 (4)	
Recurrence/metastasis			8 (11)	0 (0)	0.0008	6 (11)	0 (0)	0.0118
Local recurrence			4 (6)	0 (0)	0.0194	4 (7)	0 (0)	0.0416
Distant metastasis			5 (7)	0 (0)	0.0088	3 (5)	0 (0)	0.0791
Average follow-up period (months, mean ± SD)			79 ± 23	75 ± 23	0.1148	79 ± 24	75 ± 24	0.0983

*CRC* colorectal carcinoma, *VM* vertical margin, *SD* standard deviation, *tub* tubular adenocarcinoma, *pap* papillary adenocarcinoma, *por* poorly differentiated adenocarcinoma, *sig* signet-ring adenocarcinoma, *muc* mucinous adenocarcinoma, *LNM* lymph node metastasis

### Prognosis after additional surgery

The average follow-up periods in the VM distance < 500 µm group and VM distance ≥ 500 µm group were 79 ± 23 and 75 ± 23 months, respectively. The rates of recurrence and metastasis in the VM distance < 500 and ≥ 500 µm groups after PSM were 6 (11%) and 0 (0%) of 55, respectively. The rate of local recurrence and distant metastasis in the VM distance < 500 µm groups after PSM were 4 (7%) and 3 (5%) of 55, respectively. The rate of overall recurrence and local recurrence were also significantly higher in the VM distance < 500 µm than that in the ≥ 500 µm group after PSM (Table 2).

**Fig. 2:**
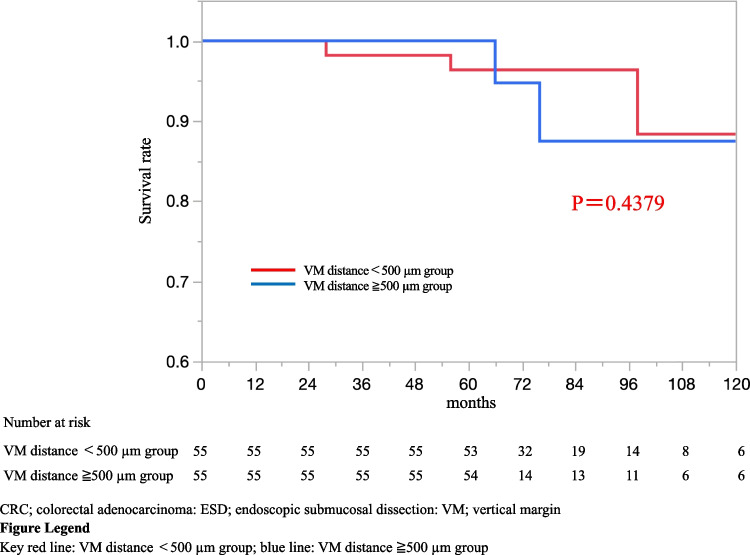
Kaplan–Meier curves for overall survival rate of patients with T1 CRC underwent additional surgery after propensity-score matching (*n* = 110)

**Fig. 3:**
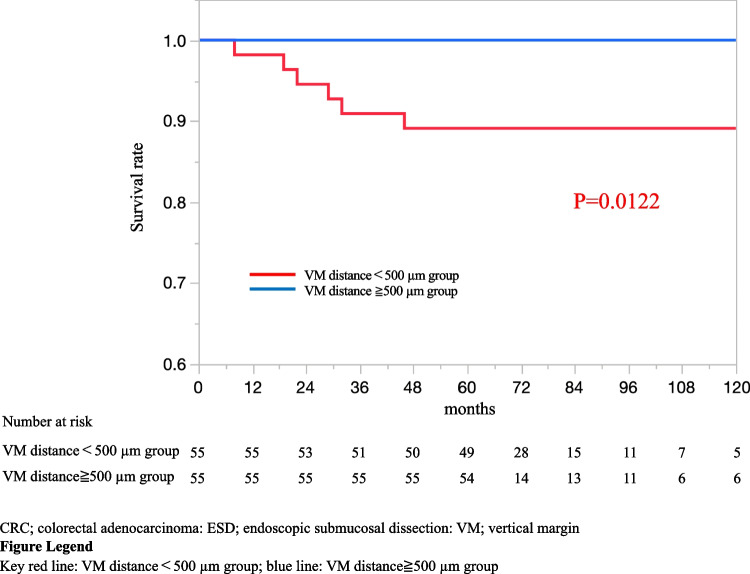
Kaplan–Meier curves for recurrence-free survival rate of patients with T1 CRC underwent additional surgery after propensity-score matching (*n* = 110)

Table 3 shows the characteristics of eight patients with recurrence. All patients had recurrence within 5 years of ESD. The recurrence sites were as follows: intrapelvis (*n* = 1 [0.6%]), pelvic lymph node (*n* = 2 [1.1%]), lung and pelvic lymph node (*n* = 1 [0.6%]), lung (*n* = 2 [1.1%]), liver (*n* = 1 [0.6%]), and liver and lymph node (*n* = 1 [0.6%]). Two patients of them had LNM positive after additional surgery. Seven recurrences were located in the rectum. Six of eight recurrences were positive for lymphatic invasion or venous invasion. Five recurrences had tumor budding grade 2 or 3. All patients with recurrence were included in the VM distance < 500 µm groups, and two patients had VM positive. In patients with T1 CRC (SM invasion depth of ≥ 1000 µm) who underwent additional surgery after ESD, the 5-year OS rates in the VM distance ≥ 500 µm group and VM distance < 500 µm group were 100 and 96.3%, respectively (*p* = 0.437; Fig. 2), whereas that of RFS significantly differed at 100 and 89.0%, respectively (*p* = 0.012; Fig. 3).

**Table 3:** Characteristics of 8 patients with recurrence

Case	Age (years)	Sex	Tumor location	Tumor size (mm)	Macroscopic type	SM depth (µm)	Ly/V	Main histology	Histology in invasive front	BD	VM distance (µm)	Recurrence site	Interval of recurrence (months)	Prognosis
LNM negative														
1	76	M	Ra	65	0-IIa	6000	− / +	Well	Well	1	290	Distance	19	Death (from other disease)
2	73	F	Ra	60	0-Is	5700	− / +	Pap	Mode	2	210	Distance	29	Death (from other disease)
3	6	M	Rb	40	0-IIa + IIc	2000	+ / +	Mode	Por	3	380	Distance Local	46	Alive
4	73	F	Rb	25	0-Is	2800	+ / +	Well	Mode	2	130	Local	22	Alive
5	62	M	A/C	30	0-IIa	3300	+ / +	Well	Mode	2	VM positive	Distance	53	Alive
6	64	M	Rb	20	0-Is + IIc	2800	+ / −	Well	Mode	2	450	Local	32	Alive
LNM positive														
7	56	M	Rb	35	0-Is	7000	+ / −	Mode	Mode	1	400	Local	8	Death (from original disease)
8	69	M	Rb	7	0-Is	1220	+ / +	Well	Well	1	VM positive	Distance	5	Death (from original disease)

*SM* submucosal, *Ly* lymphatic invasion, *V* venous invasion, *BD* budding grade, *VM* vertical margin, *Ra* rectum above the peritoneal reflection, *Rb* rectum below the peritoneal reflection, *A/C* ascending colon, *Well* well-differentiated tubular adenocarcinoma, *Pap* papillary adenocarcinoma, *Mode* moderately differentiated tubular adenocarcinoma, *LN* lymph node

## Discussion

This multicenter study found that ESD for T1 CRC required complete en bloc resection, including sufficient SM, to reduce the risk of recurrence after additional surgery.

The pathological risk factors for LNM include SM invasion depth ≥ 1,000 µm, an unfavorable histological grade (poorly differentiated adenocarcinoma or mucinous carcinoma or signet-ring cell carcinoma), positive lymphatic and/or venous invasion, and tumor budding grade 2/3 according to the JSCCR guidelines [3, 5, 9, 17]. Furthermore, others have found that female sex, left-sided colorectal lesions, rectal lesions, and completely disrupted muscularis mucosae are risk factors for LNM [5, 14, 17–19]. The LNM rate of T1 CRC range is 9‒15.8% [3, 4, 7, 8, 18, 21–26]. In other words, additional surgery for all cases with risk factors for LNM might result in unnecessary or excessive treatment, which is a concern.

To select patients with low risk factors for LNM, a specimen that can be properly evaluated is needed. We previously reported that en bloc ESD, as a total excisional biopsy for clinical T1b CRC, is effective and establishes a precise histological diagnosis [11]. However, ESD for T1b tumors is technically difficult, and some lesions cannot be resected en bloc*.* We previously reported that SM fibrosis or poor differentiation at the deepest invasive front of the tumor is associated with a high risk of positive VM in ESD for CRC with SM deep invasion [12]. In addition, Yasue et al. reported that pathological T1b CRC with obvious depression and severe fibrosis have a high risk of incomplete VM ESD [27]. Patients with VM positive require additional surgery because the likelihood of recurrence is high due to local remnants of cancer [3]. Moreover, residual tumors and incomplete ER are associated with high risk for local recurrence [20, 21]. Growth of residual tumors after ER is reportedly more rapid [28]. Therefore, very careful ESD is needed for T1b CRC with preoperative suspicions of obvious depression, mucus components at the deepest point on endoscopic ultrasonography (EUS), or the expectation of severe SM fibrosis.

Several studies have reported whether ER before surgery affects the subsequent prognosis in patients with T1 CRC. The local recurrence rate of pathologically high-risk T1 CRC after ER is 2.7–20.1% [7, 23, 25, 26, 29], while that for additional surgery after ER is 0–2.5% [23, 25, 29–32]. Additional surgery deters local recurrence of T1 CRC with high-risk factors for LNM. Moreover, prior ER does not affect the recurrence or prognosis of T1 CRC after additional surgery [29, 32, 33]. We previously found no significant differences in 5-year OS (96.9% vs. 92.0%) and 5-year disease-free survival rates (96.7% vs. 98.6%) after treatment between the additional surgery after ESD and surgery alone groups, respectively [29]. Therefore, prior ESD for T1 CRC had no adverse oncological effects on en bloc histological resection. However, whether additional surgery would be effective in patients with VM-positive cases is unclear because these reports included and evaluated both VM-positive and VM-negative cases [29, 32, 33]. Kono et al. reported remnant cancer in the intestinal tract resected by additional surgery in one case with VM distance of < 500 µm and in one case with positive VM [34]. In addition, Belderbos et al. reported that a positive resection margin was an independent risk factor for recurrence [20]. Thus, a positive VM in ESD might also be a risk factor for recurrence. We previously evaluated associations between the VM distance and recurrence or metastasis and the prognosis of patients with T1b CRC. We found that the 5-year OS and disease-specific survival rates were significantly lower in the VM distance < 500 µm group compared with the VM distance ≥ 500 µm group [14]. This study had similar results, although ER was limited to cases resected by ESD.

These results do not apply to all patients with T1 CRC, but rather to patients with T1b CRC (SM invasion depth of ≥ 1000 µm) who underwent additional surgery after ER.

Why recurrence or metastasis was more prevalent in the VM distance < 500 µm group was higher was under discussion. Several opinions have been offered to explain this phenomenon. During colorectal ESD, tumor cells are exfoliated into the intestinal lumen [35]. Colorectal cancer cells might become implanted into artificial ulcers after ER [36]. Neoplastic cells might shed from the tumor surface and become implanted on the exposed SM layer or directly into the damaged lymphatics of an artificial ulcer because ESD for primary lesions is a lengthy process [36]. Moreover, for stage I CRC, the cause of recurrence is either undetectable local residual tumors or the presence of micrometastasis [37]. Several studies reported that lymphatic invasion is an independent risk factor for metastatic recurrence in patients with surgically treated pT1 CRC [38–41]. To predict for the risk for LNM, Kajiwara et al. performed a multivariate analysis of a logistic regression analysis and developed a nomogram that incorporated SM invasion depth, lymphatic and venous invasion, predominant histological grade, sex, and location, which were found to be independent risk factors for LNM [42]. Ichimasa et al. developed an artificial intelligence model by analyzing 45 variables including pathological risk factors and serum biomarkers for preoperative detection of LNM in patients with T1 CRC; their model significantly reduced unnecessary additional surgery compared with the JSCCR guidelines without missing the patients with LNM [43]. One report on predictive models for LNM showed a strong association between LNM and recurrence [44]. By applying these nomograms and artificial intelligence models, a predictive nomogram of the risk for recurrence would be established. We believe that including the VM distance as a nomogram parameter may increase the ability to predict risk for recurrence in future models. Complete en bloc resection with a sufficient SM layer (VM distance ≥ 500 µm) by ESD for T1 CRC is essential to reduce the risk of recurrence.

Predicting whether T1 CRC is sufficiently distant from the VM to be resected by ESD is important. Preoperative EUS is useful for predicting the VM distance [12, 45]. We defined the distance from the tumor invasive front to the muscle layer on EUS as being tumor-free and found that classifying tumor-free distance as < 1 or ≥ 1 mm was useful for preoperative prediction of VM distance ≥ 500 µm [45]. In the near future, preoperative EUS will become increasingly important for evaluating whether pT1b CRC can be treated by ESD as a total excisional biopsy.

This study has several limitations. First, this was a retrospective cohort study based on clinical records. Second, we re-evaluated the pathological diagnosis and features, including tumor budding; however, we did not re-evaluate lymphovascular invasion using immunohistochemical staining in all cases. Therefore, assessment of lymphovascular invasion may have been underestimated. Third, we could not collect data about parameters such as performance status and comorbidities. At least 12 LNs should be dissected to accurately diagnose advanced colon cancer according to the National Comprehensive Cancer Network, and some patients with 12 LNs were dissected and evaluated for LNM; thus, LNM might have been underestimated. Fourth, the sample size of this study was relatively small, so there may have been effects from the other factors despite adjusting for the effects of various covariates by propensity-score matching. Large multicenter studies are needed to overcome these limitations.

## Conclusions

The recurrence rate was significantly higher in the VM distance < 500 µm group compared with the VM distance ≥ 500 µm group, and the 5-year OS and RFS rates were significantly lower in the VM distance < 500 µm group than in the VM distance ≥ 500 µm group after PSM. Therefore, ensuring a sufficient VM distance during ESD is important to reduce the risk of recurrence after additional surgery.

## Supplementary Information

Below is the link to the electronic supplementary material.

Supplementary file1 (DOCX 33 KB)

## Data Availability

No datasets were generated or analysed during the current study.
